# Supplementary material for: Chromothripsis during telomere crisis is independent of NHEJ, and consistent with a replicative origin
Source: Genome Res. 2019 May;29(5):737–49. doi: 10.1101/gr.240705.118 (PMC6499312; doi:10.1101/gr.240705.118)
Supplement: Supplemental Material [file supp_gr.240705.118_Supplemental_file_1.zip › contigs/annotated_contigs/DB45/contig.2.DB45_length_680_mean_cov_11.5529411765.docx]

**DB45_length_680_mean_cov_11.5529411765**

```
CACGTTCTTGACTGGGACAAAAAGCCAAAAATTAGGAACCAAAAAAGTCATTGAAAAAGAGAAGTTAAAATAGGGAGCTCTAGAAAATG
 >chr8:139039964-139040284 + E=9e-181
CTCATAAACAGAGTGGGGTGCTGACTGCCTCAGTAGAGAAAAGGCTGATAACTGTATCAGGCTAGCCAAACAGGAGAACATTAGGATAG

AGCATTGTCACCAAGGATAATTTAGAAAGTATAAGACAAATCAAGCCAGTGGCGTTATCCCAACATTATAAAGGCAAAGTCCATTTTAG

AAGCAGCCTAATTCCTGAAACTTACACTTCTTTCCTGAAAGCTGTCACTGTCT|ACTA|CATAAGTAGTATTATGTGGAAAGTCTTTCA
                                                          >chr8:138844650-138845006 - E
AAAGTATTAAGTTCAGATTTAAAAACATTTAGGATTGCCTGCTTGCTGACCAGCCCCAGGATAGGATGCTGGGGTTACAAAGGATAATA
=2e-202
AAACATGGGTTCTATTCTCTAGCAGCTTATGACCTGCTATGAAAGTATCACCAACAGGTTAGAGTCTGTTAAGTGTTAAGAGAAAGGTA

AACATGGTTCTATGGGAATTCCAAATGGCCCAGTTTCAAGTTTGAGGGTTATAAAGTTTGCCCCAGGAATCTCTCATGAATTCCATGTG

AATTAGACAAGACCTTGTGTATGGAAGTGTTTTATAAATATGTGATGAGAGACATAGCT
```
